# Aging perceptions matter for the well-being of elderly Turkish migrants, especially among the chronically ill

**DOI:** 10.1186/s12877-018-0902-4

**Published:** 2018-09-26

**Authors:** Jane M. Cramm, Anna P. Nieboer

**Affiliations:** Department of Socio-Medical Sciences, Erasmus School of Health Policy & Management (ESHPM) of the Erasmus University Rotterdam (EUR), P.O. Box 1738, 3000 DR Rotterdam, The Netherlands

**Keywords:** Turkish, Aging perceptions, Well-being, Older people, Ageing, Consequences of aging, Feeling in control, Emotional representations, Social production function, Chronic diseases

## Abstract

**Background:**

Research on cultural ideology with respect to aging perceptions leading to poorer health and well-being is necessary to improve the quality and effectiveness of (preventive) healthcare delivery in reaching immigrant elderly people and delivering care tailored to their needs. Despite the potential benefits of positive aging perceptions on well-being, there is a lack of empirical quantitative research on aging perceptions among elderly Turkish migrants. Therefore, the current study aimed to identify the importance of aging perceptions for the well-being of Turkish elderly in Rotterdam.

**Methods:**

The current research is a large-scale quantitative study aimed at investigating the contribution of aging perceptions to well-being among elderly Turkish migrants in Rotterdam. All Turkish people aged > 65 years were identified using the Rotterdam municipal register and invited to participate in the study. In total, 680 Turkish respondents returned completed questionnaires (32% response rate).

**Results:**

The average respondent age was 72.90 (SD, 5.02) (range, 66–95) years and approximately half of the respondents (47.6%) were women. The majority of the respondents was of a low education (80.3%) and reported a low income level (83.4%). The mean number of chronic diseases among study participants was 2.68 (SD, 1.87) (range, 0–10). Being female (*p* ≤ 0.01), being single (*p* ≤ 0.01), having a low education level (*p* ≤ 0.01) and number of chronic diseases (*p* ≤ 0.001) were negatively associated with well-being. In addition, negative perceptions on aging were negatively associated with well-being while positive perceptions on aging were positively associated with well-being. Stepwise regression analyses showed a mediating effect of perceptions of aging on the relationship between the number of chronic diseases and the well-being of study participants.

**Conclusions:**

Aging perceptions, especially perceived *consequences* of aging (both positive and negative), feelings of *control* (both positive and negative), and *emotional representations* are important to the well-being of Turkish elderly residing in the Netherlands. These results indicate the importance of the development of interventions in the perceptions on aging in the elderly Turkish population in Western Europe.

## Background

The number of elderly is rising rapidly. In the Netherlands, the proportion of elderly immigrants is expected to increase at a greater rate (by 163%) than natives (by 44%) in the years between 2009 and 2025 [1]. The majority of immigrants in the Netherlands live in large cities [2] with Turkish people being the largest group of immigrants [3]. Poor physical and mental health [4, 5], chronic diseases [6], functional limitations, and health care utilization [7] are more prevalent among immigrant populations (including the Turkish) than among natives, indicating that immigrants are a particularly vulnerable group.

Perceptions of aging are known to influence the well-being and coping strategies among the elderly [8–13]. Furthermore, positive aging perceptions are positively correlated with longevity [14–16] and reduce feelings of anxiety and depressive symptoms [17, 18]. Barker and colleagues [17] identified *timeline*, *consequences*, *control* and *emotional representation* as critically important aging perceptions affecting the well-being of Ireland natives, and Slotman and colleagues [10] identified the same among the elderly in the Netherlands. *Timeline* refers to the extent to which awareness of the aging process is either chronic (e.g. I am always aware of the fact that I am getting older) or cyclical (e.g. I go through cycles in which my experience of aging is more positive or negative). Such perceptions have been found to affect health [19]. *Consequences* of aging refers to the impact of aging on one’s life across a variety of domains which can either be positive (e.g. as I get older I appreciate things more) or negative (e.g. getting older makes everything a lot harder for me). Prior research has shown that *consequences* of aging are associated with well-being (positive *consequences*) and depression (negative *consequences*) [20]. *Control* concerns beliefs about the ability to change or influence the aging process. *Control* can be positive (e.g. whether getting older has positive aspects depends on me) or negative (e.g. slowing down with age is not something I can control). Those who have a strong sense of control can more easily increase feelings of well-being throughout life [21]. *Emotional representations* refer to negative emotions such as anxiety, fear, worry and sadness (e.g., I get depressed when I think about getting older). Such negative emotions are known to cause health decline [22].

Given the many benefits of positive perceptions on aging, the development of interventions with an emphasis on altering negative perceptions is crucial [23]. Moreover, such intervention is especially necessary among vulnerable groups such as elderly Turkish migrants living in large cities. Deterioration of health and well-being is consistently found to be significantly higher among immigrant populations compared to native Dutch people. Research on cultural ideology with respect to aging perceptions leading to poorer health and well-being is necessary to improve the quality and effectiveness of (preventive) healthcare delivery in reaching immigrant elderly people and delivering care tailored to their needs. Despite the potential benefits of positive aging perceptions on well-being, there is a lack of empirical quantitative research on aging perceptions among elderly Turkish migrants. Therefore, the current study aimed to identify the importance of aging perceptions for the well-being of Turkish elderly in Rotterdam.

## Methods

### Data collection

The Rotterdam municipal register was used to identify all community-dwelling Turkish people aged > 65 years residing in Rotterdam. Of the 2350 people identified respondents, 213 were ineligible due to change of address (*n* = 110), serious medical issues or death (*n* = 102), or non-Turkish ethnic background (*n* = 1) leading to a final sample of 2137 respondents. These respondents were asked to participate in a questionnaire via mail followed by a postal reminder. All mailings were written in both Dutch and Turkish. If no response was obtained, a home visit was made (minimum of two attempts per home). The above procedure led to a total of 680 respondents who completed the questionnaires (response rate of 32%).

Data collection took place between March 2015 and February 2016, with the exception of summer months (March – June 2015) given that most Turkish people spend their time in Turkey during that period.

### Ethical approval

According to the Central Committee on Research Involving Human Subjects (CCMO), the current study did not fall within the scope of the Medical Research Involving Human Subjects Act and therefore it was not necessary to undergo prior review by an accredited Medical Research and Ethics Committee or the CCMO. All respondents were informed about the aims of the study and that the study was anonymous and voluntary prior to providing consent to participate.

### Measures

#### Well-being

Well-being was measured with the 14-item Turkish version of the Social Production Function Instrument for the Level of Well-being short [SPF-ILs] [24]. The stimulation item: “Are your activities challenging to you?” on the original 15-item Dutch version [25] proved problematic during validation and thus was omitted from the Turkish version. The SPF-ILs measures levels of physical (comfort, stimulation) and social (behavioral confirmation, affection, status) well-being. Examples of questions are: “Do people really love you?” (affection), “Do you feel useful to others?” (behavioral confirmation), “Are you known for the things you have accomplished?” (status), “In the past few months have you felt physically comfortable?” (comfort), and “Do you really enjoy your activities?” (stimulation). Responses were given on a four-point scale ranging from never (1) to always (4), with higher mean scores indicating greater well-being. A total score was calculated based on the mean of the five subscales. Cronbach’s alpha of the SPF-ILs based on the five subscales was 0.76, indicating good reliability.

#### Perceptions of aging

The 21-item Aging Perceptions Questionnaire–Short version (APQ-S) was used to assess aging perceptions among Turkish elderly [10, 17]. The APQ-S assesses aging perceptions across seven (sub) dimensions as identified by Barker and colleagues [17] (as described in the introduction). The seven dimensions are (1) chronic awareness of the aging timeline, (2) cyclical awareness of the aging timeline, (3) positive experiences with the consequences of aging, (4) negative experiences with the consequences of aging, (5) positive feelings about being in control of the aging process, (6) negative feelings about not being in control of the aging process, and (7) negative emotional reactions toward aging. Each dimension has three items, with responses ranging from 1 (‘totally disagree’) to 5 (‘totally agree’). The items of the *control negative* dimension were reverse coded, so that higher scores indicated more perceived control.

#### Background characteristics

Respondents were asked to report the highest educational level completed in the Netherlands or abroad and were also given the option to check off ‘no schooling’ or to write down other, unlisted forms of schooling. This variable was dichotomised into completion of elementary school or less (low) and more than elementary school (high).

Income level was determined based on the respondent’s reported monthly household income, including social benefits, pensions, and alimony. Responses ranged from 1 (‘less than €1,000 a month’) to 4 (‘€3,050 a month or more’). ‘Do not know/do not want to tell’ was included as a fifth category. Income level was dichotomised into low (less than €1350) and high (€1350 or more).

Respondents were asked to indicate whether they were married, divorced, widowed, single, or cohabitating. A dichotomous variable was created: divorced, single, or widowed were included in one category and married or cohabitating in the other category.

The questionnaire also solicited information on age, gender and number of chronic conditions. The number of chronic conditions was assessed by asking respondents to report the number of chronic conditions they experienced in the past 12 months. A list of 14 chronic conditions (e.g., lung diseases, cardiovascular diseases, diabetes) was used in addition to a blank space to list other conditions. Only conditions that were classified as chronic by O’Halloran, Miller, and Britt [26] were included.

### Analyses

Sample characteristics were examined for the study sample. Bivariate associations among all variables were calculated to investigate the relationships between background characteristics, aging perceptions and well-being. Finally, regression analyses were performed to identify the relationship between aging perceptions and well-being while controlling for background characteristics.

## Results

Table 1 displays the descriptive statistics for all independent variables and scores on well-being. Of the 680 respondents, the average age was 72.90 (SD, 5.02) (range, 66–95) years and 47.6% were women. The majority of respondents was of low education (80.3%) and reported a low income level (83.4%). The mean number of chronic diseases among study participants was 2.68 (SD, 1.87) (range, 0–10).

**Table 1:** Descriptive statistics of respondents (*N* = 680)

Demographic characteristics	Range	% or mean (SD)
Sex (female)		47.6%
Age (years)	66–95	72.90 (5.02)
Marital status (single/widowed)		28.7%
Education (low)		80.3%
Income (low)		83.4%
Number of chronic diseases	0–10	2.68 (1.87)
Timeline (negative awareness of aging)		
Chronic/acute	1–5	3.78 (0.92)
Cyclical	1–5	3.53 (0.75)
Perceived consequences of aging		
Positive	1–5	3.58 (0.98)
Negative	1–5	3.84 (0.94)
Feeling in control		
Control over positive ageing effects	1–5	3.45 (0.98)
Control over negative ageing effects	1–5	2.25 (0.86)
Negative emotional representations	1–5	2.87 (1.03)
Well-being	1–4	2.78 (0.55)

Correlations of independent variables and well-being are displayed in Table 2. The results of bivariate analyses showed that being female (*p* ≤ 0.01), being single (*p* ≤ 0.01), having a low educational level (*p* ≤ 0.01) and number of chronic diseases (*p* ≤ 0.001) are negatively associated with well-being. Negative aging perceptions (*timeline* chronic and cyclical, negative *emotional representations* and negative *consequence*) were all negatively associated with well-being. A positive relationship was found between the positive aging perceptions (positive *control* and positive *consequences*) and well-being. No significant relationship was found between the aging perception of negative *control* and well-being.

**Table 2:** Associations among background characteristics, aging perceptions, and well-being (*N* = 680)

	1	2	3	4	5	6	7	8	9	10	11	12	13
1. Sex (female)													
2. Age	−.05												
3. Marital status (single)	0.41***	0.14***											
4. Education (low)	0.24***	0.05	0.12***										
5. Income (low)	0.18***	0.06	0.14***	0.23***									
6. Number of chronic diseases	0.28***	0.01	0.16***	0.19***	0.12**								
7. Chronic/acute (timeline)	0.14***	0.09*	0.07	0.24***	0.08	0.25***							
8. Cyclical (timeline)	0.02	0.04	0.02	0.08	0.04	0.21***	0.40***						
9. Positive consequences	−0.04	− 0.06	− 0.05	− 0.02	0.01	− 0.12***	0.10*	0.12**					
10. Negative consequences	0.09*	0.10**	0.07	0.18***	0.08	0.29***	0.45***	0.46***	− 0.00				
11. Control positive	− 0.10**	− 0.10**	− 0.09*	−0.11**	− 0.02	− 0.25***	− 0.05	−0.03	0.51***	−0.02			
12. Control negative	−0.02	−0.06	− 0.05	−0.10*	0.05	−0.13***	− 0.37***	− 0.38***	− 0.11**	−0.41***	− 0.02		
13. Negative emotional representations	0.03	0.07	0.07	0.07	−0.02	0.21***	0.23***	0.50***	−0.08*	0.43***	−0.09*	−0.38***	
14. Well-being	−0.11**	− 0.06	− 0.10**	−0.11**	− 0.06	−0.27***	− 0.14***	−0.15***	0.29***	−0.24***	0.39***	0.02	−0.29***

Notes: ***p ≤ 0.001; **p ≤ 0.01; *p ≤ 0.05 (two-tailed)

The results of the stepwise multivariate analyses are displayed in Table 3. In step 1, background variables were entered into the analysis. There was a significant negative correlation between the number of chronic diseases and well-being among elderly Turkish migrants (β = − 0.26; *p* < 0.001). In step 2, aging perceptions were entered into the analysis and the relationship between number of chronic conditions and well-being was no longer significant. The strength of the association dropped from β = − 0.26 to β = − 0.09, indicating a mediating effect of perceptions of aging on the relationship between the number of chronic conditions and the well-being of elderly Turkish migrants. In addition, while *timeline* aging perceptions were significantly related to well-being in the correlational analyses, the relationship was lost in the multivariate analysis. There was a significant association between well-being and perceived *consequences* of aging, feeling in *control* and *emotional representations*. There was a significant negative correlation between negative perceptions and well-being and a significant positive correlation between positive perceptions and well-being. Although the bivariate analyses showed no significant relationship between negative *control* and well-being, in the multivariate analyses they are significantly related.

**Table 3:** Results of stepwise regression analysis

Characteristic	Well-being		Well-being	
	β (SE)	*p*	β (SE)	*p*
Sex (female)	0.01 (0.05)	0.856	−0.01 (0.05)	0.818
Age (years)	−.008 (0.01)	0.064	−0.03 (0.00)	0.503
Marital status (single/widowed)	−0.01 (0.06)	0.914	0.01 (0.05)	0.892
Education (low)	−0.07 (0.06)	0.126	−0.02 (0.06)	0.648
Income (low)	−0.04 (0.07)	0.349	−0.04 (0.06)	0.273
Number of chronic diseases	−0.26 (0.01)	< 0.001	−0.09 (0.01)	0.052
Timeline (negative awareness of aging)				
Chronic/acute			0.02 (0.03)	0.696
Cyclical			−0.05 (0.04)	0.329
Perceived consequences of aging				
Positive			0.11 (0.03)	0.012
Negative			−0.22 (0.03)	< 0.001
Feeling in control				
Control over positive ageing effects			0.25 (0.03)	< 0.001
Control over negative ageing effects			−0.15 (0.03)	0.001
Negative emotional representations			−0.15 (0.03)	0.002
Adjusted R^2^	8%		26%	

## Discussion

Turkish migrants are among the most disadvantaged elderly groups in European society [27] and as such it is critical to understand factors related to their well-being to promote healthy aging. The current study revealed that perceptions on aging play a significant role in the well-being of Turkish elderly residing in Rotterdam, the Netherlands.

After controlling for background characteristics, perceived *consequences* of aging (both positive and negative), feeling in *control* (both positive and negative) and *emotional representations* were associated with the well-being of Turkish elderly. These results are in agreement with prior research finding that health and/or well-being are significantly related to perceived consequences of aging [20], feeling in control over the positive and negative consequences of aging [21], and negative emotions concerning the aging process [22]. Also similar to earlier findings [19], the current study found a significant relationship between cyclical and chronic awareness of the aging process on well-being in the bivariate analysis. However, the association became insignificant in the multivariate analysis. The discrepancy may arise from the fact that while Westerhof and colleagues [19] investigated awareness of aging, they did not take other aging perceptions into account. In the current study, the awareness of the cyclical *timeline* of aging was significantly associated with well-being in the bivariate analysis, however, after adding *emotional representation* into the analysis, the association was no longer significant. Likewise, the significance of the relationship between acute *timeline* and well-being dissipated after negative *consequences* of aging was entered into the analysis.

The relationships between perceptions of aging and well-being were strongest with the aging dimensions feeling in control, consequences of ageing and emotional representations. While feeling in control over negative aging effects was not significantly related to well-being in the bivariate analysis it was significantly related to well-being in the multivariate analyses, but the effect was small. Finally, the two-step procedure revealed that after adding perceptions of aging into the equation, the relationship between number of chronic conditions and well-being was no longer significant, suggesting a mediating effect of aging perceptions on the relationship between the number of chronic conditions and the well-being of Turkish elderly. This is an important finding given the fact that many Turkish elderly people are dealing with chronic diseases. Interventions aimed at improving aging perceptions among chronically ill Turkish elderly may benefit their well-being.

There are limitations to the current study. First, the response rate was relatively low. However, the rate was similar to that in other surveys conducted within the same population. In addition to invitations via mail, we made a minimum of two personal contact attempts at the homes of potential participants. Prior research however, has indicated that a minimum of six contact attempts is necessary for optimal response [4]. Second, the cross-sectional design of the current study prevents the ability to draw causal inferences. However, it is hypothesized that the relationship is dynamic such that lower levels of well-being may lead to more negative perceptions of aging. Longitudinal data are needed to investigate the causal relationship between aging perceptions and well-being over time. Third, the current study was limited to Turkish elderly in Rotterdam. Moroccans are the second largest migrant group in Rotterdam and as such, a similar study among elderly Moroccans would be of interest. In addition, aging perceptions vary across cultures and as such, the study of aging perceptions among (im) migrant elderly in other countries should also be studied for comparison. Finally, we did not include aspects such as health seeking behavior or access to healthcare, which also might affect ageing perceptions as well as well-being.

## Conclusions

Aging perceptions, especially perceived *consequences* of aging (both positive and negative), feelings of *control* (both positive and negative), and *emotional representations* are important to the well-being of Turkish elderly residing in the Netherlands.

There is a trend toward an increase in the elderly Turkish population in Western Europe and it is therefore imperative to develop effective services and interventions aimed at the promotion of well-being among elderly Turkish people and more generally, implementation of immigrant-sensitive healthcare delivery. Specifically, interventions should focus on healthy age-related perceptions which have been found to be of key importance to the health and well-being of elderly people [23]. For example, Stephan and colleagues [28] found that providing favorable social comparison feedback during tasks results in more positive aging perceptions among older people. In their study, older people were asked to perform a task measuring handgrip strength and then received feedback about their performance. Those who were told that they had performed significantly better compared to others, had more positive aging perceptions and also increased their performance in a second handgrip strength measure. Miche and Wahl [29] conducted a similar study in which older people were asked to perform a cognitive task after which they received social comparison feedback. Participants were told that in the task they had performed, accuracy increases with age and most importantly, that they had made less mistakes than younger people. Participants held more positive aging perceptions after the task as compared to before the task. Taken together, the above two studies suggest increasing awareness of positive age-related changes and providing favorable social comparison feedback, is beneficial to positive aging perceptions.

In addition to positive priming (as described by the above two studies), an increase in knowledge and information about successful aging may lead to more positive aging perceptions. Wolff and colleagues [30] showed that educating older people about the positive aspects of aging and correcting negative misconceptions improves perceptions of aging. Furthermore, positive perceptions of aging and perceived control over aging-related experiences may enhance the older peoples’ abilities to cope with challenges and demands as they age.

## Abbreviations

APQ-S: Aging Perceptions Questionnaire–Short version
CCMO: Committee on Research Involving Human Subjects
SD: Standard Deviation
SPF-ILs: Social Production Function Instrument for the Level of Well-being short
